# Progress in Medicine

**Published:** 1897-09-11

**Authors:** 


### PROGRESS IN MEDICINE.

### RENAL MEDICINE.

(*Continued from page* 373.)

**Urology.**—The smegma bacillus was discovered twelve years ago, yet its deceptive resemblance to the tubercle bacillus is too often forgotten. Grünbaum[1] examined fifty specimens of urine for it, and found it in fifty-nine per cent. of the females but less frequently in that of males, though it appears to be a normal inhabitant of the male urethra. However, it can be entirely avoided by careful catheterisation either in males or females, and though it and the tubercle bacilli stain under exactly the same reagents, a distinction can be made from the circumstance that the smegma bacilli in urine are alone decolourised by one minute's immersion in absolute alcohol. They also very rarely show the irregularities in contour of the tubercle one, and are usually found in groups or on shed epithelial cells. Weichselbaum and others recommend decolourising with alcohol for five minutes and upwards. [See The Hospital, p. 315, vol. xxi.]

The colon bacillus appears occasionally in urine when no cystitis or kidney disease exists, or, at least, a bacillus which resembles it. The mode of its entrance into the bladder is doubtful when no inflammatory conditions are present, though Escherich thinks that in the case of women it may enter through the urethra. Not infrequently it is the concomitant of an important form of cystitis, especially in young children. Frumpp[2] describes twenty-nine cases, of which nineteen were severe with extreme wasting, strangury, and sometimes hæmaturia, ascending nephritis, and death.

The urine may be cloudy, acid, slightly fœtid, and in bad cases may contain much albumen, leucocytes, and peptones, as well as casts. He finds patients recover best when salol is given and the bladder washed out with lysol, though mild cases mend without any treatment. Baginsky[3] found this bacillus in several cases of pyelonephritis in children, sometimes accompanied by the proteus, pyocyaneus, and bacillus lactis. How far the colon bacillus was to be regarded as the cause was uncertain. The urine was at times normal, and at others contained albumen and pus-cells. The temperature varied from subnormal, with sharp rises at times. The changes in the urine from time to time may be easily overlooked, but when noticed give a clue to the disease even in mild cases. In addition to dyspeptic symptoms there may be constipation, diarrhœa, or membranous colitis; multiple abscesses may form in the kidneys and necrotic foci. Thus it would seem that in early life many obscure cases of kidney disease may be recognised by the occasional passage of albuminous urine containing pus and the bacillus coli.

The amœba coli, again, is sometimes found in cases of hæmaturia. Wijnhoff[4] and others have recorded attacks of strangury and colic in such cases, the protozoa being passed chiefly at the time of these painful paroxysms.

Achard[5] has employed his test for the existence of doubtful kidney lesions in some fifty additional cases. It is carried out by injecting a centimetre of a five per cent. solution of methylene blue into the gluteal muscles, and noting the rapidity with which it is eliminated by the kidneys, and thus shows the permeability of the kidneys, or their failure to excrete. Where there is a localised lesion, and the rest of the kidney is healthy, there may be no delay, and in some cases of epilepsy there is at first an excretion of a colourless chromogen, which gives a green colour on heating with acetic acid, instead of the methylene blue. In epithelial nephritis there appears to be no delay, but in other forms of kidney failure the rapidity of elimination seems to be a useful test in the absence of definite symptoms of disease.

An interesting paper by W. K. Fyffe[6] deals with the existence of albumoses in the urine. Most cases of so-called peptonuria are really due to albumose, which is a bye-product of the action of bacteria, and important as showing their presence. Peptone is shown to exist in urine by neutralising, and then saturating with ammonium sulphate, which precipitates it, but not albumose. The latter, on the other hand, forms a ring with cold nitric acid, which disappears on heating. Thus the two bodies can be distinguished. In phthisis with large cavities and much breaking down of tissue, in severe rheumatic fever, in uræmic convulsions, and in ulcerative endocarditis albumose may be present, as well as in pneumonia and after anti-toxin injections in diphtheria. Generally speaking its appearance betokens grave danger, though not always the formation of pus. When it is replaced by albumen during the course of pneumonia, Fyffe foretells a fatal issue, but its occurrence after anti-toxin is not serious, and may be regarded as a normal elimination of the bacterial products.

Hæmatoporphyrin has only been recognised of late years, but it occurs in various diseases, such as phthisis, acute rheumatism, and lead poisoning. G. Parker[7] reports a case following a second attack of lead poisoning. The patient had apparently recovered, but three days after the onset of influenza, from which others suffered as well, she developed colic and wrist-drop. Ten days later hæmatoporphyrin appeared with severe abdominal pain. Lead was found in the urine, but the source was undetermined, and marked paralysis took place. A later attack was fatal, with renewed excretion of the hæmatoporphyrin. T. McCall Anderson[8] reports another instance where it was found with annual attacks of hydroa-æstivale, which had recurred for more than twenty years, leaving troublesome cicatrices. The patient's brother, a young fisherman from Stornoway, suffers from the same eruption, and passes urine of the same colour during the attacks. The pigment seems to be an alkaline hæmatoporphyrin which yields the acid pigment on treatment with sulphuric acid. Nakarai[9] lays down that lead poisoning is always accompanied by the excretion of these pigments. They are also found in sulfonal intoxication and intestinal hæmorrhage, but rarely in other disorders.

In a discussion on lactosuria, McCann,[10] from the results of 100 cases, states that sugar is normally present in the urine of women during lactation, and at some period in every case, but usually in largest quantities on the fourth or fifth days of the puerperium. The amount varies with the quantity and quality of the milk and the suckling of the child, the average being about one and a half grains per ounce. Another abnormal constituent is urobilin, which has been recently noted by Rolleston,[11] after trional had been given for sleeplessness to a patient who had suffered from heart disease, with nutmeg liver and slight jaundice. As this substance is allied to hæmatoporphyrin, he thought it probable that the trional was the cause of it appearing; but A. E. Garrod believes that it has a different mode of origin, being often caused by the action of bacteria on the bile pigment in the intestine. If this latter substance is retained unduly in the intestines the conversion easily takes place. R. Stein[12] advocates the use of sulphosalycylic acid as a valuable test for albumen. He adds a few crystals to filtered urine. If albumen is present a uniform cloudy opalescence appears. The urine should be acid, and as all proteids are precipitated it is as well to boil the liquid, when any precipitate due to peptones or albumose disappears. One great advantage is that it is unaffected by mucin, if present in small quantities. He claims for it also that it is much more exact than the hot or cold nitric acid tests, and even better than that by heating urine acidulated with acetic acid and that with cyanide of potash. Moreover, there is no resolution if an excess of the acid is added. The cause of the toxicity of urine is little understood, but facts as to its production are being gradually accumulated. Casciani[13] finds that a vegetable diet reduces it, and if this diet is habitual, and the fatigue endured is slight, the toxicity is very low indeed. It increases generally with the amount of exercise and of meat consumed. After severe burns, the urine often contains pyridin and similar bodies, which are supposed by Fränkel and Spiegler to be formed from the proteids split up by the heat. The substance seems to be highly poisonous, and is said to be associated under these circumstances with cysteine and other derivatives from the half-burned proteids.

Klemperer[14] continues his advocacy of urea in renal lithiasis and dropsy, hepatic or cardiac, where the kidneys are sound. In some cases the amount of diuresis is immense, five litres of urine being passed in 24 hours. He gives 10 grammes daily for two days, then 15 grammes for two more days. If no effect is seen it will be useless to continue, but otherwise he goes on until 200 or 250 grammes, or even more, have been taken. It appears to prevent attacks of renal colic and lumbar pain where gravel or gout exists. Klemperer administers it as a powder with equal parts of soda and prepared chalk, but it can be given equally well dissolved simply in water.

The extensive use of injections of saline fluid with or without simultaneous venesection in uræmia, and the good results obtained, have led to investigations as to the best kind of fluid. Edes[15] mentions that for two years a modification of Ringer's fluid has been used in Massachusetts General Hospital. It is made by adding 0·1 gramme of Ca. Cl. and 0·75 gramme of K Cl. to 1,000 cc. normal salt solution. This has been given chiefly by intravenous injection in quantities of 500 to 2,000 cc. The fluid can be made up, placed in Florence flasks, and then sterilised to keep till required, the flasks being placed in hot water when wanted. The object of the additional salts is to obtain a better action of the heart, and the effects of the injection seem more lasting than those of simple salt solution.

[1] Lancet, Jan. 9. [2] Med. Rec., Mar. 20. [3] Brit. Med. Journ., July 17. [4] Amer. Journ. Med. Sciences, April. [5] Med. Week, June 25. [6] Australas. M. Gazette, May 20. [7] Bristol Med. Ch. Journ., p. 213, June. [8] Scottish Med. S. Journ., Feb. [9] Med. Rec., Ap. 24. [10] Lancet, April 24. [11] B. Med. Journ., March 20. [12] Med. Rec., Jan. 16. [13] La Riforma Med., xii., 147. [14] Med. Week, Jan. 8. [15] Boston Med. and S. J., March.

